# Astrocytic Interleukin-15 Reduces Pathology of Neuromyelitis Optica in Mice

**DOI:** 10.3389/fimmu.2018.00523

**Published:** 2018-03-19

**Authors:** Zhiguo Li, Jinrui Han, Honglei Ren, Cun-Gen Ma, Fu-Dong Shi, Qiang Liu, Minshu Li

**Affiliations:** ^1^Department of Neurology, Tianjin Neurological Institute, Tianjin Medical University General Hospital, Tianjin, China; ^2^Center for Neuroinflammation, Beijing Tiantan Hospital, Capital Medical University, Beijing, China; ^3^Shanxi University of Traditional Chinese Medicine, Taiyuan, China; ^4^Department of Neurology, Barrow Neurological Institute, St. Joseph’s Hospital and Medical Center, Phoenix, AZ, United States

**Keywords:** astrocytes, complement-dependent cytotoxicity, neuromyelitis optica-IgG, IL-15, neuromyelitis optica

## Abstract

Astrocyte loss induced by neuromyelitis optica (NMO)-IgG and complement-dependent cytotoxicity (CDC) is the hallmark of NMO pathology. The survival of astrocytes is thought to reflect astrocyte exposure to environmental factors in the CNS and the response of astrocytes to these factors. However, still unclear are how astrocytes respond to NMO-IgG and CDC, and what CNS environmental factors may impact the survival of astrocytes. In a murine model of NMO induced by intracerebral injection of NMO-IgG and human complement, we found dramatic upregulation of IL-15 in astrocytes. To study the role of astrocytic IL-15 in NMO, we generated a transgenic mouse line with targeted expression of IL-15 in astrocytes (IL-15^tg^), in which the expression of IL-15 is controlled by a glial fibrillary acidic protein promoter. We showed that astrocyte-targeted expression of IL-15 attenuates astrocyte injury and the loss of aquaporin-4 in the brain. Reduced blood–brain barrier leakage and immune cell infiltration are also found in the lesion of IL-15^tg^ mice subjected to NMO induction. IL-15^tg^ astrocytes are less susceptible to NMO-IgG-mediated CDC than their wild-type counterparts. The enhanced resistance of IL-15^tg^ astrocytes to cytotoxicity and cell death involves NF-κB signaling pathway. Our findings suggest that IL-15 reduces astrocyte loss and NMO pathology.

## Introduction

Neuromyelitis optica (NMO) is a severe autoimmune disease in the central nervous system that predominantly affects the optic nerves and spinal cord (1, 2). Binding of NMO-IgG to water channel aquaporin-4 (AQP4), primarily expressed at the end-feet of astrocytes, initiates complement-dependent cell cytotoxicity (CDCC) on astrocytes, followed by blood–brain barrier breakdown, recruitment of granulocytes and macrophages and cytokine secretion, which result in secondary oligodendrocyte injury, demyelination, and neuronal injury (3–6). In NMO disease, death of astrocytes is thought to be pivotal because it initiates a cascade of inflammatory responses that further exacerbate CNS injury. Astrocytes could play an active role in regulating CNS inflammation and brain homeostasis *via* producing inflammatory mediators, energy and oxidative precursors, as well as scavenging extracellular cytotoxic substances in a variety of neurological diseases (7–9). Although it has long been known that astrocytes are targets of NMO-IgG and CDC, how astrocytes respond to NMO-IgG and CDC are still poorly understood.

The survival of astrocytes depends on their exposure and receptiveness to CNS environmental factors. Interleukin-15 is a proinflammatory cytokine that impacts the homeostasis and intensity of immune response in autoimmune diseases. In the periphery, studies have demonstrated that IL-15 contributes to the immunopathology of several inflammatory diseases, such as rheumatoid arthritis and inflammatory bowel disease (10, 11). In the CNS, IL-15 is minimally expressed in physiological conditions, but the level of IL-15 in the brain is upregulated after CNS injuries. Astrocytes have been found as a major source of IL-15 in the CNS after injuries (12–16). Previous studies suggest that IL-15 would either aggravate or attenuate inflammation and neural injuries depending on timing, disease stage and types (17, 18). However, the role of IL-15 in NMO pathology remains unknown. In a murine model of NMO, we found that IL-15 is dramatically upregulated in astrocytes. To test the potential role of astrocytic IL-15 in NMO, we generated a transgenic mouse line with targeted expression of IL-15 in astrocytes (IL-15^tg^ mice) and examined NMO pathology in these mice.

## Materials and Methods

### Mice

The study was performed in accordance with the National Institutes of Health Guide for the Care and Use of Laboratory Animals. All experimental procedures were approved by Animal Care and Use Committees of Barrow Neurological Institute (AZ, USA) and Tianjin Neurological Institute (Tianjin, China). IL-15^tg^ mice were developed by Genetically Engineered Mouse Models Core at Univercity of Arizona (Tuscon, AZ, USA), the method of IL-15^tg^ mice development and genotype identification was done as previously described (13–15). IL-15^tg^ mice were backcrossed to the C57BL/6 background for at least 10 generations before experiments were performed. All mice were kept in specific pathogen-free conditions and kept at a standard 21°C with a 12 h light/dark cycle at the animal facility of the Barrow Neurological Institute or Tianjin Neurological Institute.

### NMO Animal Model Procedure

Weight-matched adult female mice, aged 8–12 weeks old IL-15^tg^ mice and WT littermates, were used in our experiments. NMO mouse model is induced by intra-parenchymal injections of NMO-IgG [recombinant antibody (rAb-53)], AQP4-specific IgG, which is cloned from intrathecal plasma cells in early NMO (19) and human complement (HC). In brief, mice were anesthetized by inhaling 3.5% isoflurane and maintained by inhalation of 1.0–2.0% isoflurane in 70% N_2_O and 30% O_2_ by a face mask, then mounted in a stereotactic frame. A midline scalp incision was made to expose bregma and lambda, a burr hole was made 2 mm to the right of the bregma. A 26-gauge needle attached to 10 μl gas-tight glass syringe (Hamilton) was inserted 3-mm deep to infuse 8 μl PBS containing 2 μg NMO-IgG (rAb-53) and 3 μl HC to the parenchymal tissue at a rate of 0.5 μl/min. During the entire procedure, rectal temperature was maintained at 37°C with a heating lamp.

### Neuroimaging

Neuromyelitis optica lesions and BBB permeability were detected using a 7T small animal, 30-cm horizontal-bore magnet and BioSpec Advance III spectrometer (Bruker, Billerica, MA, USA) with a 116-mm high power gradient set (600 mT/m) and a 72-mm whole-body mouse transmit/surface receive coil configuration. T2-weighted images were acquired at day 3 after NMO induction. Scan parameters and T2-weigthed acquisition were as we described previously (13–15, 20). Axial 2D multi slice T2-weighted images of brain were acquired with TR = 4,000 ms, TE = 60 ms, FOV = 19.2 mm × 19.2 mm, matrix 192 mm × 192 mm. In order to assess BBB permeability, the post-contrast T1 was obtained 10 min after the administration of gadopentetate dimeglumine (Gd-DTPA) (Magnevist) with dosage of 0.2 mmol/kg bodyweight, as described (13–15, 21). Axial 2D multi slice T1-weighted images of brain were acquired with TR = 322 ms, TE = 10.5 ms, FOV = 28 mm × 28 mm, matrix 256 mm × 256 mm. During MRI scan, the animal’s respiration was continually monitored by a small animal monitoring and gating system (SA Instruments) *via* a pillow sensor positioned under the abdomen. Mice were placed on a heated circulating water blanket (Bruker) and the normal body temperature (36–37°C) was maintained. The MRI data were analyzed with Image J software (NIH).

### Cell Isolation and Flow Cytometry

Quantitative analysis of immune cell subsets and cell apoptosis were prepared from brain tissue and stained with fluorochrome-conjugated antibodies as described (13–15, 22). At day 3 after NMO, we isolated single cell suspension from the brain. Briefly, mice were deeply anesthetized and brain was removed immediately after perfusion with PBS. For CNS immune cell infiltration, brain tissue was cut into small pieces and digested with 1 mg/ml collagenase in 10 mM Hepes/NaOH buffer at 37°C for 1 h. The cell pellet was re-suspended in 70% percoll, then overlaid with 30% percoll. After centrifugation, the cell monolayer between 30 and 70% percoll interface was harvested as mononuclear cells. For astrocyte and cell death analysis, we harvested the brain and homogenized with 70 μm nylon cell strainers in PBS, then, we removed the myelin using 30% percoll with the centrifuge at 700 *g* for 10 min. The cell pellet was collected for analysis of Caspase3 and IL-15 expression.

We used flow cytometry to analyze leukocyte subsets and neural cell apoptosis. Single cell suspensions were stained with antibodies and appropriate isotype controls. All antibodies were purchased from BD Bioscience or eBioscience, Inc. unless otherwise indicated. The procedure of cell staining followed the manual protocol. The following antibodies were used: CD3 (145-2C11), NK1.1 (PK136), CD8 (53-6.72), CD45 (30-F11), CD11b (M1/70), CD4 (GK1.4), Ly6G/C (RB6-8C5), glial fibrillary acidic protein (GFAP) (GA5), Caspase 3 (3G2). Flow cytometric data were acquired on a FACSAria flow cytometer (BD Bioscience) and analyzed with Flow Jo software version 7.6.1.

### Immunofluorescence and H&E Staining

Mice were terminally anesthetized and perfused with PBS followed by 4% PFA. Brains were removed and embedded in paraffin. 5-μm thick coronal sections were deparaffinized and rehydrated in serial ethanol. For immunofluorescence staining, sections were permeabilized with 0.3% TritonX-100 for 15 min, then incubated with blocking solution consisting of 5% donkey serum, followed by incubating with antibodies against GFAP (Abcam), AQP4 (Santa Cruz), Claudin5 (Life Tech) at 4°C overnight. After washing with PBS, slices were incubated with appropriate fluorochrome conjugated secondary antibodies: donkey anti-rabbit 488 (Invitrogen), donkey anti-goat 594 (Invitrogen), donkey anti-rabbit 546 (Invitrogen), respectively, at room temperature for 1 h. Finally, all slices were incubated with fluoro-shield mounting medium with DAPI (Abcam). Images were taken with a fluorescence microscope (model BX-61, Olympus). To get the image with the whole lesion in each slice, we took 10–15 visual fields in a row under a × 40 field of microscope around the lesion site (GFAP and AQP4 loss), then merged these images into a bigger one using the photoshop7.0 software. For H&E Staining, tissue sections were stained with hematoxylin and eosin. Images were taken with a microscope (model BX-61, Olympus). The data were quantified using ImageJ.

### Primary Astrocytes Culture

Primary cortical astrocytes were prepared as previously described (13–15). Briefly, mixed cortices were removed from 1 to 3 days old WT and IL-15^tg^ pups and minced with scissors in ice-cold HBSS (Gibco), then digested with 0.25% trypsin solution (Gibco) at 37°C for 30 min. The dissociated cells were rinsed and re-suspended in high glucose DMEM and counted. Cells were plated in 35-mm culture dishes at a density of 2.5 × 10^4^ cells/cm^2^. After 2 days, the medium [High glucose DMEM (Gibco) + 10% heat-inactivated FBS (Gibco) + 1% Penicillin/Streptomycin (Gibco)] was changed to remove cell debris. 7–8 days later, we passaged the first split astrocyte population at the appropriate cell concentration for the experiment. The purity of astrocytes was up to 95%.

### Complement–Dependent Cytotoxicity (CDC)

Cultured astrocyte were plated onto 96-well microplates at 20,000 cells/well and grown at 37°C/5% CO_2_ for 18–24 h. Cells were washed with PBS for three times and incubated on ice with 50 μl of rAb-53 for 30 min in DMEM (Gibco). Thereafter, 2.5 μl pooled normal HC serum (Innovative Research) was added to the cells and cultured for additional 60 min at 37°C. Cytotoxicity was measured by lactate dehydrogenase (LDH) release assay using a commercial kit (Sigma) according to the manufacturer’s instructions (23). LDH release from cells was calculated as a percentage of total LDH in each sample.

### Western Blot

Cells were lysed with cell lysis buffer (Cell Signaling Technology) containing protease inhibitor cocktail (Sigma-Aldrich) and phosphatase inhibitor mixture (Sigma-Aldrich) for 30 min on ice. The samples were centrifuged at 14,000 rpm for 15 min at 4°C to remove cell debris. Protein concentration was determined using a BCA protein assay kit (Pierce). Proteins were subjected to SDS-PAGE gel (Bio-Rad) and transferred to a PVDF membrane (Millipore). The membrane was blocked with 5% non-fat milk in TBS solution containing 0.05% Tween-20 for 1 h at room temperature. Then the membrane was incubated with antibodies against GAPDH (1:1,000), IκB (1:500, Cell signaling pathway), and pIκB (1:500, Cell signaling pathway) overnight at 4°C. After washing three times with TBST solution, the membrane was incubated with HRP-conjugated goat anti-rabbit (1:2,000; Zymed) for 1 h at room temperature. Immuno-reactive bands were detected using enhanced chemiluminescence (Thermo Scientific) and captured with an Odyssey Fc Imager (Li-cor biosciences Inc.). Western blot data were analyzed with ImageJ software.

### Statistics

We determined each sample size by power analysis using a significance level of α = 0.05 with 80% power to detect statistical differences. SAS 9.1 software (SAS Institute Inc., Cary, NC, USA) was used for power analysis and sample-size calculations. All values are expressed as mean ± SE. Statistical data analyses were performed using Graphpad Primes 6 software. Two-tailed unpaired Student’s *t*-test was used to analyze the statistical significance of two groups. Where appropriate, One-way ANOVA were used for three or more groups. Values of *p* < 0.05 will be considered significant.

## Results

### Astrocyte-Targeted Expression of IL-15 Is Upregulated in NMO Mice

**Figure 1:**
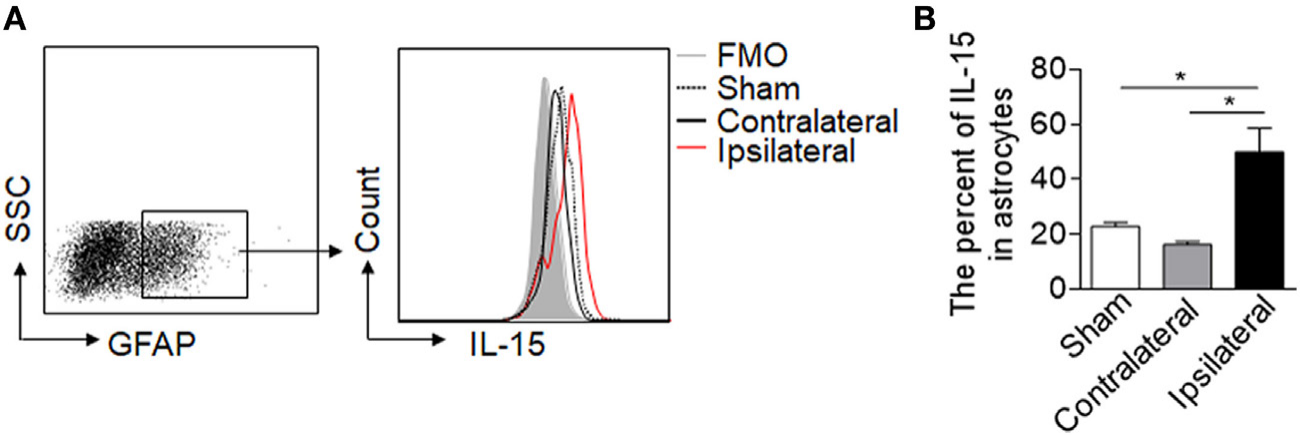
Upregulation of IL-15 in astrocytes after neuromyelitis optica (NMO) induction. Mouse brains were injected with 2 μg rAb-53 plus 3 μl human complement. At day 3 after NMO induction, the brain tissues were harvested for flow cytometry analysis. **(A,B)** The data show expression of IL-15 in astrocytes from the tissue of the ipsilateral and contralateral hemisphere, as well as sham control, *n* = 4 mice/group. Data represent the mean ± SE, *p < 0.05.

Astrocytic IL-15 is inducible and has a dual role on brain injury in different CNS diseases (17, 18), to assess whether astrocytic IL-15 level was increased in NMO, we compared the expression of IL-15 in astrocytes in the brain tissue from the ipsilateral hemisphere to the contralateral hemisphere or sham control at day 3 after injection of recombinant NMO-IgG (rAb-53) and HC. Flow cytometry data show that the amount of IL-15 in astrocytes is much higher after NMO (Figures 1A,B), indicating that astrocytic IL-15 expression is related to NMO progression.

### Astrocyte-Targeted Expression of IL-15 Reduces Lesion Size in NMO Mice

To investigate the effect of astrocytic IL-15 in NMO, we adopted IL-15^tg^ mice in which IL-15 expression is controlled by a GFAP promotor, as we previously reported (13–15). The expression level of IL-15 in astrocytes is prominently higher in IL-15^tg^ mice as compared to WT littermates. Importantly, these transgenic mice develop normally without showing any abnormal behavior or infertility, and the current transgenic mice show normal nerve cell properties and immune system, no inflammatory infiltrates were seen in brain tissues of normal IL-15^tg^ mice.

**Figure 2:**
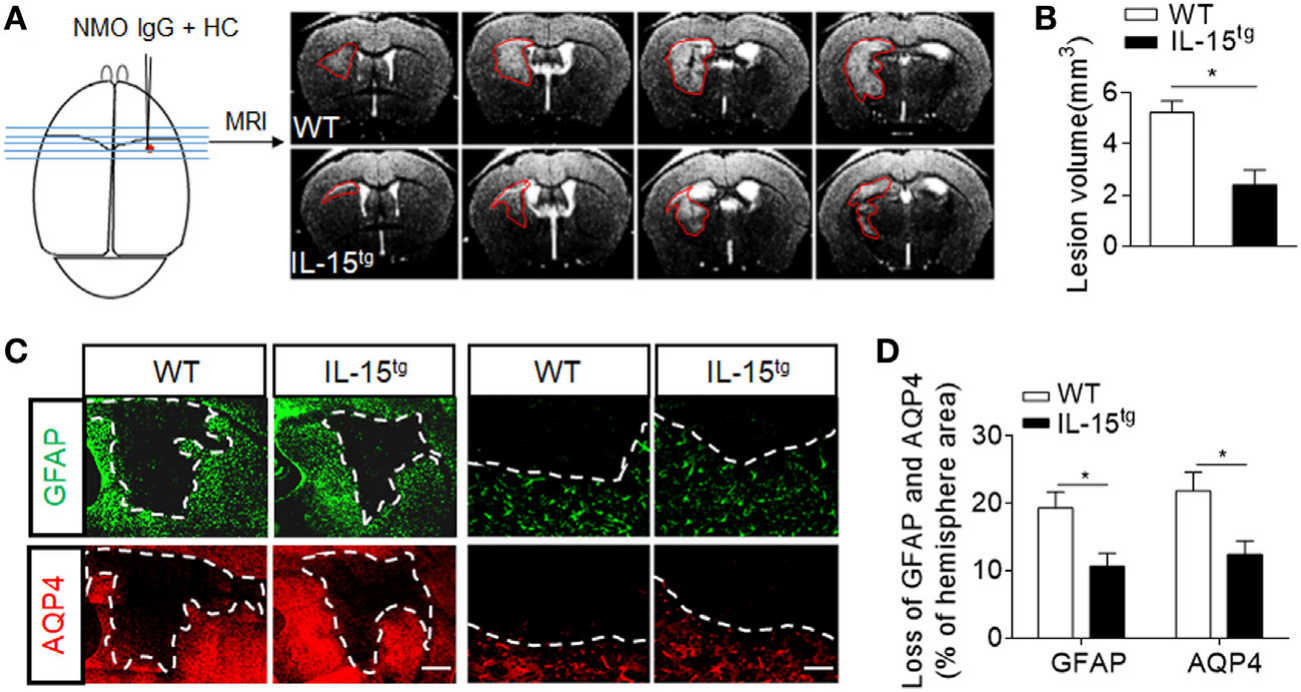
Reduced brain lesion severity and astrocytes loss in IL-15^tg^ mice subjected to injection of neuromyelitis optica (NMO)-lgG (rAb-53) and human complement (HC). Mouse brains were injected with 2 μg rAb-53 plus 3 μl HC. At day 3 after NMO induction, visualization and quantification of NMO lesion size were taken with T2-weighted images obtained with a 7 T MRI scanner. Representative of sequential MRI images of NMO lesion in the brain from WT littermates and IL-15^tg^ mice **(A)**. The left schematic view of brain denotes the injection position (red dot) and the region of brain corresponding to the MRI image in the right. Red lines in the MRI images indicate the NMO lesion area. Quantification of NMO lesion volumes in MRI images **(B)**, *n* = 12 mice/group. **(C)** Immunostaining of glial fibrillary acidic protein (GFAP) and aquaporin-4 (AQP4) in NMO lesion at day 3 after NMO induction. Image of whole lesion area (left panel) represented by loss of GFAP and AQP4 immunostaining. High magnification of immunostaining of GFAP and AQP4 around NMO lesion was shown in the right panel. White dashed line represents lesion area. Scale bar = 1mm (left panel) and 100 μm (right panel). **(D)** Quantification of the NMO lesion size with GFAP and AQP4 loss in the sections of WT and IL-15^tg^ mice, *n* = 5 mice/group. Data represent the mean ± SE, *p < 0.05.

To further understand whether astrocyte-derived IL-15 affects NMO lesion severity, we induced an animal NMO model through intracerebral injection of HC and recombinant NMO-IgG (rAb-53) in IL-15^tg^ mice and WT littermates. At day 3, 7T-MRI scans combined with conventional immunofluorescence staining for AQP4 and GFAP were used to evaluate NMO lesion size. MRI data show that the group of IL-15^tg^ mice have markedly reduced NMO lesion size as compared to WT littermates (IL-15^tg^ vs WT: 2.40 ± 0.58 vs 5.23 ± 0.45 mm^3^) (Figures 2A,B), corresponding to less loss of GFAP and AQP4 immunostaining in the region of injection site (IL-15^tg^ vs WT: 9.00 ± 2.1% vs 17.24 ± 1.9%) (Figures 2C,D). A higher magnification of the lesion shows the characteristics of AQP4 and GFAP loss and reactive astrocytes existing around the lesion area (Figure 2C). These results indicate that astrocytic IL-15 may attenuate brain injury after NMO.

### Astrocyte-Targeted Expression of IL-15 Attenuates BBB Leakage and Tight-Junction Loss in NMO Mice

**Figure 3:**
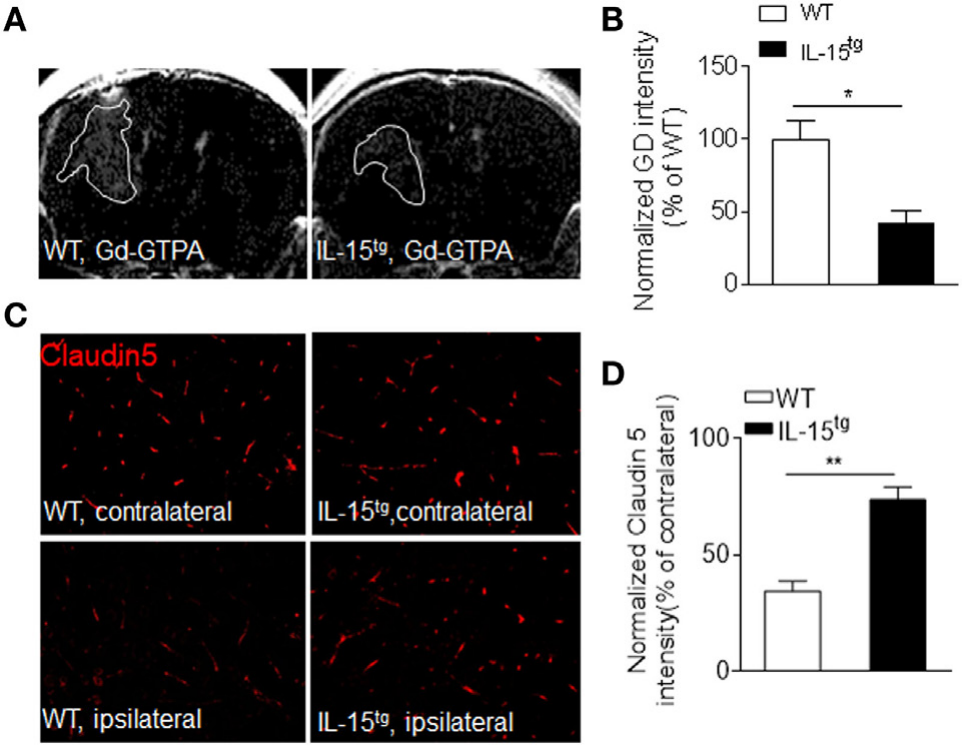
Reduced blood–brain barrier permeability in IL-15^tg^ mice subjected to neuromyelitis optica (NMO) induction. **(A,B)** T1-weighted MRI were performed to visualize and quantify Gd-DTPA leakage into the brain parenchyma in the indicated groups at day3 after NMO induction. White line indicates Gd-GTPA leakage area. **(C,D)** Immunostaining of tight junction protein, Claudin5, in ipsilateral NMO lesion area and contralateral corresponding normal area and the quantification of the intensity. *n* = 10 sections from three mice/group. Data represent the mean ± SE, *p < 0.05, **p < 0.01.

Astrocytes, the target of NMO-IgG, are essential for the formation and maintenance of the BBB. The findings that astrocytic IL-15 attenuates astrocyte loss, prompted us to consider that BBB integrity may be preserved in IL-15^tg^ mice. As we expected, BBB permeability is more prominent in WT mice than that in IL-15^tg^ mice, as measured by T1 MRI scan (Figures 3A,B). The protein level of claudin 5, a tight junction protein, is also much higher in IL-15^tg^ mice as compared to WT littermates (Figures 3C,D). These data further supported that astrocyte-specific expression of IL-15 could prevent BBB damage.

### Astrocyte-Targeted Expression of IL-15 Reduces Brain Infiltration of Immune Cell Subsets in NMO Mice

**Figure 4:**
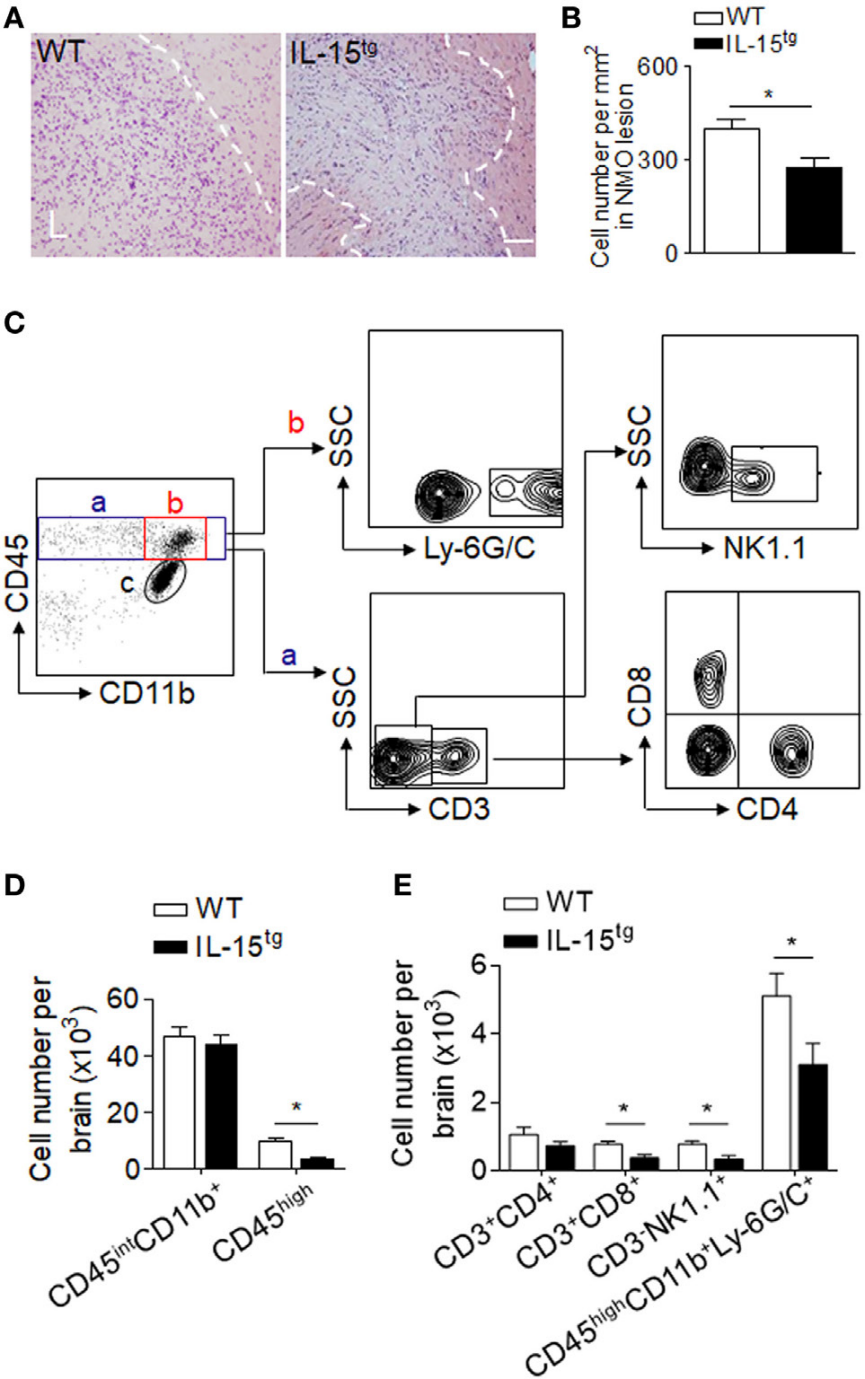
Astrocyte-targeted expression of IL-15 reduced CNS infiltration of immune cells. **(A,B)** H&E stains showed less cell density in neuromyelitis optica (NMO) lesions in IL-15^tg^ mice as compared to WT mice at day 3 after NMO induction. *n* = 5 mice/group. **(C–E)** CNS-invading immune cell subsets and microglia cell numbers were analyzed using flow cytometry at day 3 after NMO induction. Gating strategy of immune cells including macrophage and neutrophils (CD45^high^CD11b^+^ly6G/C^+^), microglia (CD11b^+^CD45^inter^), CD4^+^ T cells (CD45^high^CD3^+^CD4^+^), CD8^+^ T cells (CD45^high^ CD3^+^CD8^+^), NK cells (CD45^high^CD3^-^NK1,1^+^) **(C)**. Cell number of microglia, CNS-invading immune cells (CD45^high^), and immune cell subsets decreased in IL-15^tg^ mice as compared to WT mice after NMO induction **(D,E)**, *n* = 5 mice/group. Data represent the mean ± SE, *p < 0.05.

Injection of NMO-IgG and HC in mouse brain produces marked inflammation. Leukocyte infiltration and microglia activation contribute to the brain inflammation in NMO development. IL-15, as an inflammatory cytokine, has functions on a wide range of immune cells. To investigate the inflammation and immune response in the brain of WT and IL-15^tg^ mice, we used H&E staining (Figures 4A,B) and flow cytometry to detect the subsets of leukocytes, as well as microglia, the gating strategy as in Figure 4C. We did not observe a significant difference in the number of CD45^int^/CD11b^+^ (microglia) in the brain between WT and IL-15^tg^ mice after NMO induction. In contrast, accumulation of CD45^high^ and Ly6G/C (macrophage and neutrophil), the prominent immune cells in lesions of patients with NMO, was markedly decreased in IL-15^tg^ mice (Figure 4D). In addition, IL-15^tg^ mice exhibited significantly reduced cell numbers of CD3, CD8, and NK cells in the brain as compared to WT littermates (Figure 4E). These data show that IL-15^tg^ mice have extensively decreased leukocyte infiltration in the brain compared to WT littermates, indicating that the contribution of astrocytic IL-15 to brain inflammation is likely dependent on BBB damage and astrocyte loss.

### IL-15 Promotes the Survival of Astrocytes From CDC-Induced Cell Death

**Figure 5:**
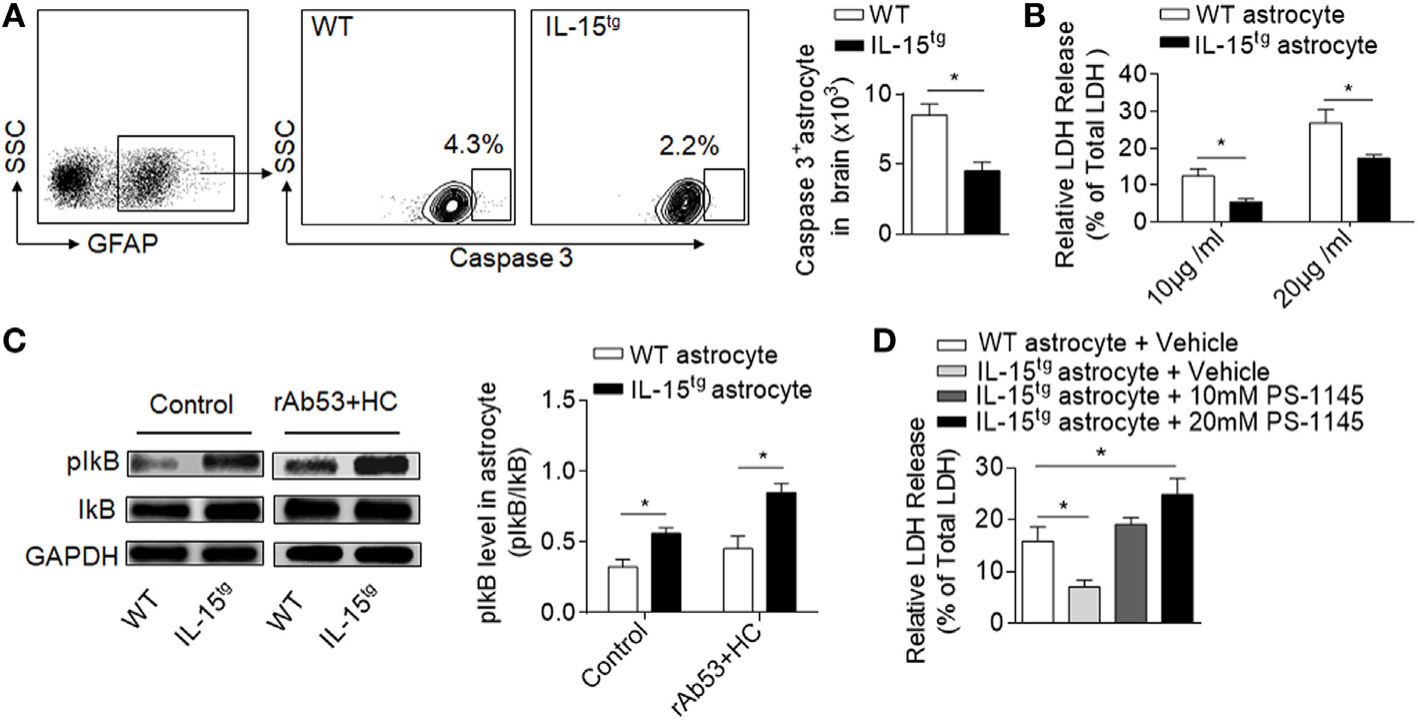
IL-15 protects astrocyte from CDC-mediated astrocyte injury. **(A)** Gating strategy of astrocytes expressing Caspase 3 and quantification of cell number of Caspase 3^+^ astrocytes in WT and IL-15^tg^ neuromyelitis optica (NMO) mice at day 3 (*n* = 5 mice/group). **(B)** Quantification of lactate dehydrogenase (LDH) release in cultured astrocytes isolated from WT littermates and IL-15^tg^ mice incubated with recombinant NMO-IgG (rAb-53) (10 or 20 μg/ml) and 5% human complement (HC) for 60 min, cell viability was determined by LDH assay. Data represent the mean (±SE) of three independent experiments. **(C)** Western blot were performed to test activation of NF-κB signaling pathway in astrocytes treated with 10 μg/ml rAb-53 and 5% HC for 60 min, pIκB values are relative to IκB (three independent experiments). **(D)** PS-1145, a NF-κB pathway inhibitor, could attenuate the protection of astrocytic IL-15 on CDC-mediated cell damage *in vitro*. Data represent the mean (±SE) of three independent experiments, *p < 0.05.

CDC-mediated astrocyte injury is the key event during NMO development, which initiates secondary inflammation and NMO lesion development. Our findings that astrocytic IL-15 reduces astrocyte loss and BBB damage indicate that IL-15 may have the function of inhibiting astrocyte injury induced by CDC. To answer this question, we analyzed the cell apoptosis of astrocytes in the brain after NMO induction. We found that both the percent and cell number of astrocytes expressing caspase3 is significantly reduced in IL-15^tg^ mice, suggesting that astrocytic IL-15 may have a function of resistance to CDC-mediated cell death (Figure 5A). While, there is no significant difference of astrocyte apoptosis in the contralateral hemisphere between WT and IL-15^tg^ mice after NMO (data not shown). To further confirm the protective role of astrocytic IL-15, we incubated primary cultured astrocytes from the brain of WT and IL-15^tg^ mice with 10 or 20 μg/ml NMO-IgG and 5% HC respectively. Cell viability was measured by LDH assay. The data show that cell death of astrocytes from IL-15^tg^ mice were reduced as compared to that from WT littermates when cultured with 10 or 20 μg/ml NMO IgG (Figure 5B). These findings demonstrate that IL-15 might be a survival factor for astrocytes from CDC induced by NMO-IgG and HC.

### IL-15 Protects Astrocytes Against CDC *via* NF-κB Signaling Pathway

NF-κB pathway has been reported to be responsible for nucleated cells resistant to CDC-dependent cytotoxicity (24). To determine whether NF-κB is involved in CDC resistance of astrocytic IL-15, we compared the protein levels of phosphorylated IκB in astrocytes. The data show that the level of pIκB is much higher in astrocytes from IL-15^tg^ mice than in WT littermates when incubated with NMO-IgG with HC (Figure 5C), suggesting that IL-15 or NMO-IgG binding could activate NF-κB signaling pathway. To further demonstrate whether NF-κB is responsible for CDC resistance, we treated astrocytes with PS-1145, which specifically blocks NF-κB activation by inhibiting phosphorylation of IκB. The data show that pretreatment of PS-1145 make the astrocytes from IL-15^tg^ mice more sensitive to complement-dependent necrosis (Figure 5D). These results together suggest that NF-κB pathway may contribute to the protective role of astrocytic IL-15 on CDC resistance after NMO.

## Discussion

The current study provides the first evidence that astrocyte-derived IL-15 can protect against NMO pathology. As documented here, astrocytic IL-15 can be a survival factor to attenuate astrocytes loss induced by CDC, together with attenuated BBB injury and CNS inflammation. IL-15 augments the survival of astrocytes against CDC *via* NF-κB signaling. In all, our results reveal IL-15 as a key factor to reduce astrocyte loss and CNS inflammation in NMO.

The finding that IL-15 is upregulated in astrocytes in NMO mice suggest that astrocytes can respond to NMO-IgG and CDC and modulate CNS inflammation. This is consistent with previous reports showing that astrocytes undergo dramatic changes of immune profiles including upregulation of chemokines and cytokines after the binding of NMO-IgG (25, 26). In particular, blockade of these factors could reduce cytotoxicity and preserve AQP4 protein level in cultured astrocytes (25). In contrast to the proinflammatory factors reported in this study, we found that astrocytic IL-15 reduces NMO severity and CNS inflammation, suggesting that astrocytes could play an active role in NMO pathology.

IL-15 is an inflammatory cytokine with a function on a wide range of immune cells (27, 28). Much evidence demonstrated that IL-15 can boost immune cells to exacerbate disease progression such as rheumatoid arthritis and stroke (13–15, 29). However, there are still documents, which demonstrated that IL-15 is beneficial to experimental autoimmune encephalomyelitis (17, 18). In NMO mice, we found that IL-15 is dramatically upregulated in astrocytes. Using genetic manipulation, we demonstrated that astrocyte-specific expression of IL-15 could attenuate NMO pathology severity. These discrepancies between the roles of IL-15 in different CNS diseases may contribute to the setting of these diseases and the pleiotropic function of IL-15. In the setting of NMO, CDC-mediated astrocytes loss plays a vital role in NMO progression, which, to large extent, determines the BBB integrity and immune cell infiltration. Less astrocytes damage in IL-15^tg^ mice is likely to produce less immune cell infiltration. Additionally, it is noteworthy that IL-15 is also beneficial to neuron growth and development (30), whether IL-15 has the direct protective function on neuron survival or enhancing neurodegeneration needs further investigation.

The finding that IL-15 reduces NMO severity in mice and protects astrocytes against CDC has clinical relevance. As above described, CDC-induced astrocytes damage plays a key role in NMO pathology. As such, the use of IL-15 could be a candidate for further advanced studies to test its effectiveness to reduce NMO pathology. However, it is also noteworthy that the effect of exogenous IL-15 may be different from endogenous IL-15 as trans-presentation may be needed for the elaboration of IL-15 efficacy. In addition, the efficacy of IL-15 may vary depending on injected doses and their availability to various cell types that express IL-15 receptors. Future more advanced investigations are needed to better understand the role of IL-15 in NMO pathology. Although treatments targeting the CDC process have been investigated including complement inhibitor or NMO-IgG mutant (31, 32), no treatments are available to directly boost astrocyte survival and reduce CNS inflammation. Our results in this study suggest that IL-15 may serve as a potential therapy or at least a complementary approach to attenuate NMO pathology.

There are also limitations in this study. First, we induced NMO in mice by direct injection of NMO-lgG and HC into brain parenchyma, which may not fully mimic the scenario of pathological events in patients with NMO, such as the trigger of BBB breakdown, neuroinflammation, and peripheral immune response (33–35). Therefore, future studies are needed to verify our findings in other NMO animal models. Second, the beneficial role of IL-15 might be related to multiple cellular targets including CNS intrinsic cells and peripheral immune cell subsets. Because different doses of IL-15 may show different influences in nerve cells or immune cells, it is needed to define whether the protection of astrocytic IL-15 in NMO depends on available IL-15 concentration. Third, IL-15 receptors are widely expressed by CNS cell types such as neural progenitors. Therefore, the role of IL-15 in neuronal function or neurorepair warrant further investigations in future studies.

In conclusion, our studies provide novel insight astrocytic IL-15 in NMO pathology.

## Ethics Statement

The study was performed in accordance with the National Institutes of Health Guide for the Care and Use of Laboratory Animals. All experimental procedures were approved by Animal Care and Use Committees of Barrow Neurological Institute (Arizona, USA) and Tianjin Neurological Institute (Tianjin, China).

## Author Contributions

ML, QL, and F-DS formulated the study concept and wrote the paper. ZL, JH, and HR performed experiments. ML, ZL, and C-GM analyzed the data, interpreted the results, and assisted preparation of the manuscript.

## Conflict of Interest Statement

The authors declare that the research was conducted in the absence of any commercial or financial relationships that could be construed as a potential conflict of interest.
